# Nomogram development for predicting ovarian tumor malignancy using inflammatory biomarker and CA-125

**DOI:** 10.1038/s41598-024-66509-9

**Published:** 2024-07-09

**Authors:** Gatot Nyarumenteng Adhipurnawan Winarno, Ali Budi Harsono, Dodi Suardi, Siti Salima, Kemala Isnainiasih Mantilidewi, Hartanto Bayuaji, Ayu Insafi Mulyantari, Fajar Awalia Yulianto, Hadi Susiarno

**Affiliations:** 1 Department of Obstetrics and Gynecology, Faculty of Medicine, Universitas Padjadjaran, Dr. Hasan Sadikin General Hospital, Prof Eykman Street No 38, Bandung, 40161 Indonesia; 2 Department of Public Health, Medical Faculty, Universitas Islam Bandung, Bandung, Indonesia

**Keywords:** Ovarian tumors, Ovarian cancer, Inflammatory biomarker, CA125, Cancer, Oncology

## Abstract

Global challenges in ovarian cancer underscore the need for cost-effective screening. This study aims to assess the role of pretreatment Neutrophil-to-Lymphocyte Ratio (NLR), Lymphocyte-to-Monocyte-Ratio (LMR), Platelet-to-Lymphocyte Ratio (PLR), and CA-125 in distinguishing benign and malignant ovarian tumors, while also constructing nomogram models for distinguish benign and malignant ovarian tumor using inflammatory biomarkers and CA-125. This is a retrospective study of 206 ovarian tumor patients. We conducted bivariate analysis to compare mean values of CA-125, LMR, NLR, and PLR with histopathology results. Multiple regression logistic analysis was then employed to establish predictive models for malignancy. NLR, PLR, and CA-125 exhibited statistically higher levels in malignant ovarian tumors compared to benign ones (5.56 ± 4.8 vs. 2.9 ± 2.58, 278.12 ± 165.2 vs. 180.64 ± 89.95, 537.2 ± 1621.47 vs. 110.08 ± 393.05, respectively), while lower LMR was associated with malignant tumors compared to benign (3.2 ± 1.6 vs. 4.24 ± 1.78, p = 0.0001). Multiple logistic regression analysis revealed that both PLR and CA125 emerged as independent risk factors for malignancy in ovarian tumors (P(z) 0.03 and 0.01, respectively). Utilizing the outcomes of multiple regression logistic analysis, a nomogram was constructed to enhance malignancy prediction in ovarian tumors. In conclusion, our study emphasizes the significance of NLR, PLR, CA-125, and LMR in diagnosing ovarian tumors. PLR and CA-125 emerged as independent risk factors for distinguishing between benign and malignant tumors. The nomogram model offers a practical way to enhance diagnostic precision.

## Introduction

Ovarian cancer, often known as the "silent killer," presents a significant global health challenge, marked by alarming incidence and mortality rates. According to GLOBOCAN 2020, the worldwide occurrence of ovarian cancer stood at 6.6 cases per 100,000 women, with a death rate of 4.2 per 100,000 women^1^. This disease's hidden progression often leads to late-stage diagnoses, contributing to the persistently high mortality rates. This disease's hidden progression often leads to late-stage diagnoses, contributing to the persistently high mortality rates. Even in regions with advanced screening modalities like Southern Europe, the mortality rate remains relatively high at 4.3 per 100,000 women. In developing regions such as South-East Asia, the mortality rate rises even further to a concerning 5.2 per 100,000 women^1^. The need for improved screening tests arises from the typically late presentation of the disease, which diminishes the effectiveness of treatment options and adversely affects patient outcomes.

Distinguishing between benign and malignant ovarian tumors is critically important in clinical practice as it directly impacts treatment decisions and patient prognosis. Current standard screening protocols for ovarian cancer include transvaginal ultrasound (TVUS) and risk assessment models such as the Risk of Malignancy Index (RMI) and The International Ovarian Tumor Analysis (IOTA)^2,3^. TVUS provides imaging that can help identify ovarian masses, while RMI combines Cancer Antigen 25 (CA-125) levels, menopausal status, and ultrasound findings to estimate the risk of malignancy^4^. The tests currently used for this purpose often have limitations, including variable sensitivity and specificity, and also subjective interpretation among practitioners. Furthermore, in resource-limited hospital, there is a pressing need for cost-effective and accessible methods to differentiate between benign and malignant tumors.

These challenges highlight the urgency of developing more reliable and resource-efficient diagnostic methods. The lack of a universally accepted method for early diagnosis further underscores the difficulty in detecting ovarian cancer at an early stage. This late presentation reduces the effectiveness of treatment options and adversely affects patient outcomes, emphasizing the need for improved diagnostic strategies.

CA-125 has served as the standard serum biomarker for ovarian cancer screening^5^. However, the effectiveness of CA-125 as a standalone diagnostic tool is restricted by its limited sensitivity and specificity^6^. As a result, its clinical application often requires the inclusion of additional, though costly diagnostic methods such as Magnetic Resonance Imaging (MRI) or Computed Tomography (CT) scans. Therefore, it is important to explore new diagnostic strategies that can improve the accuracy of CA-125 in distinguishing between benign and malignant ovarian tumors. Recent studies have explored the potential of combining multiple biomarkers to enhance diagnostic accuracy. For instance, integrating CA-125 with other hematological markers such as Neutrophil-to-Lymphocyte Ratio Lymphocytes, monocytes, and platelets have emerged as influential factors in the intricate interplay between the immune system and malignancies^7^. Beyond their conventional roles in immunity and hemostasis, these blood components undergo intriguing alterations in response to the presence of tumors, providing valuable insights into the potential differentiation of benign from malignant lesions. A study review reported that various measures of leukocyte quantities, such as WBC count, PLR, and NLR, have been associated with an increased risk of several types of cancer, including breast cancer, colorectal cancer, and endometrial cancer, as well as with tumor progression^8^.

The selected biomarkers, CA-125, NLR, PLR, and LMR, represent different aspects of the biological processes involved in cancer development and progression, such as tumor burden, systemic inflammation, and immune response^7^. In this study, we aim to develop a nomogram that combines CA-125 with NLR, PLR, and LMR to enhance diagnostic accuracy. This approach not only promises practicality and cost-effectiveness but also holds particular potential for regions with limited resources. The intention is to develop a nomogram that can predicting malignancy in ovarian tumor patients.

## Material and methods

### Study design and patients

All ovarian tumor patients from July 2018 to June 2023 at Dr. Hasan Sadikin Hospital were included in this retrospective study. The inclusion criteria were as follows: (1) patients with ovarian tumors, (2) who underwent histological examinations, (3) had complete laboratory data, (4) underwent surgery, and (5) had not yet received any chemotherapy. Exclusion criteria included patients with chronic or immunosuppressive diseases, secondary cancer, and incomplete data. This study was conducted after obtaining approval from the chairman of the Ethics Committee of Dr. Hasan Sadikin Central General Hospital Bandung (LB.02.01/X.6.5/298/2023), Dr. Ina Rosalina. All participants provided written informed consent, and all methods were carried out in accordance with relevant guidelines and regulations.

### Data collection

We collected demographic and clinical data, including patient age, parity, stage of malignant ovarian tumors, and histopathological results from the patients' medical records. Diagnosis of benign and malignant ovarian tumors was confirmed through histopathological analysis of the tumor tissue. Laboratory data, including pre-surgical CA125, LMR, NLR, and PLR, were obtained from the hospital laboratory database.

### Statistical analysis

Statistical analysis was performed using Stata MP version 16 for Windows. The histopathology result and IOTA conclusion was treated as a categorical variable, while age, parity, CA-125, LMR, NLR, and PLR values were considered continuous variables. Descriptive statistics were presented using frequency and percentage for categorical variables and mean ± standard deviation for continuous variables. Bivariate analysis was performed to compare the mean values of CA-125, LMR, NLR, and PLR with the histopathology results, utilizing either a t-test or Mann–Whitney test as appropriate. The cut-off point for each index test was conducted separately by judging the optimal sensitivity, specificity, and likelihood ratio (LR). Subsequently, ROC curve analysis was conducted to assess the discrimination ability and overall performance of CA-125, LMR, NLR, and PLR, using the Area Under the Curve (AUC) as a measure of diagnostic accuracy. We performed multiple logistic regression analyses to evaluate the combined diagnostic performance of NLR, LMR, PLR, and CA-125. P < 0.05 was considered to indicate a statistically significant difference.

### Ethics approval and consent to participate

This study was approved by the Ethics Committee of Dr. Hasan Sadikin Central General Hospital number LB.02.01/X.6.5/298/2023.

## Results

### Demographic and baseline data of the patients

Between July 2018 and June 2023, 221 patients with ovarian tumors were initially assessed for a study. However, 15 patients were excluded due to incomplete data, leaving 206 ovarian tumor patients for analysis. As shown in Table 1, histopathological examination revealed that 123 (59.7%) had malignant tumors, while the remaining 83 (40.3%) had benign tumors. Notably, the majority of patients with malignant tumors were diagnosed at stage III. Epithelial cell histology was the most common type observed in both malignant and benign tumors. Additionally, the average age of patients with malignant tumors was 46 years old, compared to 41 years old for those with benign tumors.

**Table 1:** Characteristics of the study patients.

Variable	Ovarian tumor		P value
	Benign	Malignant	
	N = 83	N = 123	
Age			0.05
Mean ± std	41.7 ± 13.8	45.6 ± 13.6	
Parity			0.21*
Mean ± std	2.2 ± 1.2	2.4 ± 1.2	
Origin			0.25
Epithelial	59 (71.1%)	92 (74.8%)	
Sex cord	6 (7.2%)	14 (11.4%)	
Germ cell	18 (21.7%)	17 (13.8%)	
Stage			–
I	–	33(26.9%)	
II	–	35(28.5%)	
III	–	46(37.3%)	
IV	–	9(7.3%)	
IOTA conclusion			
Benign	32(38.6%)	33 (26.9%)	0.00
Malignant	51(61.4%)	90 (73.1%)	

*Mann–Whitney Test.

The pre-surgical levels of CA-125, NLR, LMR, and PLR were analyzed in ovarian tumor patients, as presented in Table 2. NLR exhibited a significant elevation in malignant ovarian tumors compared to benign cases (5.56 ± 4.8 vs. 2.9 ± 2.58, P = 0.0001). Similarly, LMR was significantly lower in malignant ovarian tumors compared to benign tumors (3.24 ± 1.6 vs. 4.24 ± 1.78, p = 0.0001). Furthermore, PLR displayed a substantial increase in malignant ovarian tumors compared to benign tumors (278.12 ± 165.2 vs. 180.64 ± 89.95, P = 0.0001). CA125 levels were significantly higher in malignant ovarian tumors compared to benign tumors (537.2 ± 1621.47 vs. 110.08 ± 393.05, P = 0.0001).

**Table 2:** Comparison of pre-treatment NLR, LMR, PLR, and CA125 with histopathology findings of the patients.

		Benign	Malignant	P Value
NLR	Mean ± Std	2.9 ± 2.6	5.6 ± 4.8	0.0001*
LMR	Mean ± Std	4.2 ± 1.8	3.2 ± 1.6	0.0001*
PLR	Mean ± Std	180.6 ± 89.9	278.1 ± 165.2	0.0001*
CA125	Mean ± Std	110.0 ± 393.0	537.2 ± 1621.4	0.0001*

NLR, neutrophil-to-lymphocyte ratio; LMR, lymphocyte-to-monocyte-ratio; PLR, platelet-to-lymphocyte ratio.

*Mann–Whitney test.

### Bivariate analysis for NLR, LMR, PLR, and CA-125 in distinguishing benign and malignant ovarian tumors

**Figure 1:**
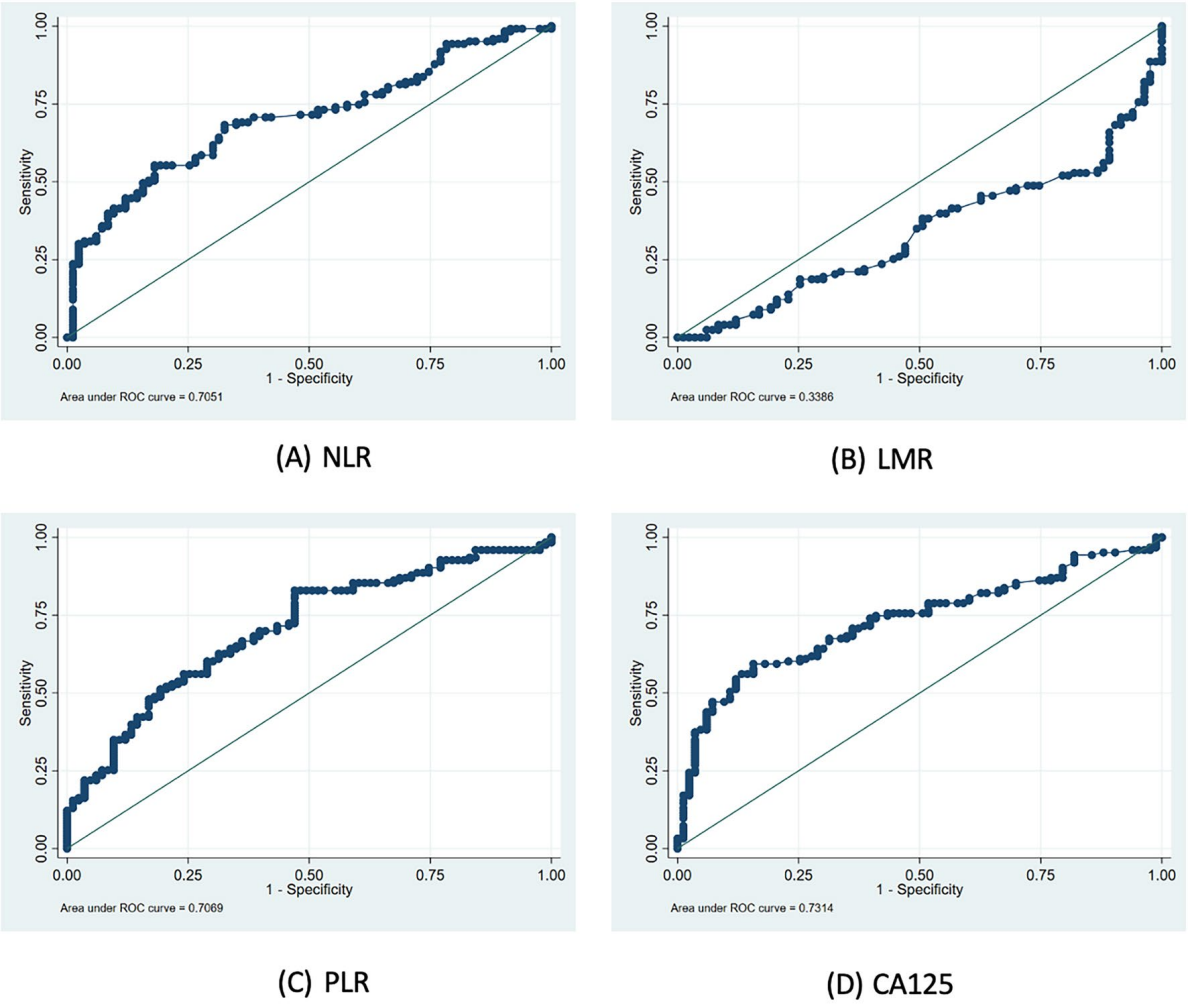
Receiver operating characteristic (ROC) curves for inflammatory biomarkers and CA125 in predicting ovarian tumor malignancy. (**A**) ROC curve for Neutrophil-to-Lymphocyte Ratio (NLR) with an AUC of 0.7051. (**B**) ROC curve for Lymphocyte-to-Monocyte Ratio (LMR) with an AUC of 0.3386. (**C**) ROC curve for Platelet-to-Lymphocyte Ratio (PLR) with an AUC of 0.7069. (**D**) ROC curve for Cancer Antigen 125 (CA-125) with an AUC of 0.7314.

Table 3 shown the analysis of optimal cut-off points for NLR, LMR, PLR, and CA125 as their diagnostic performance in distinguishing between benign and malignant ovarian tumors. Notably, NLR with a cut-off value of ≥ 2.72 demonstrated a sensitivity of 68.29% and specificity of 67.47%. The likelihood ratio (LR) for NLR was calculated at 2.099293, indicating a moderate discriminative capacity. The AUC in the ROC analysis for NLR was 70.51%, suggesting a fair overall diagnostic accuracy as shown in Fig. 1. Conversely, LMR exhibited a cut-off value of ≥ 3.67, accompanied by a sensitivity of 41.46% and specificity of 43.37%. The LR for LMR was notably low at 0.732121, indicating a limited discriminative ability. The AUC in the ROC analysis further confirmed these findings, with LMR displaying the lowest value at 33.86%, underscoring its suboptimal performance in distinguishing between benign and malignant ovarian tumors. PLR with a cut-off value of ≥ 174.1, exhibited a sensitivity of 65.04% and specificity of 65.06%. The LR for PLR was calculated at 1.861477, indicating a moderate discriminative capacity. The AUC in the ROC analysis for PLR was 70.69%, suggesting a fair overall diagnostic accuracy. Meanwhile, CA-125 with a cut-off value of ≥ 78, demonstrated a sensitivity of 66.67% and specificity of 68.67%. The LR for CA-125 was 2.153846, suggesting a moderate discriminative capacity. The AUC in the ROC analysis for CA-125 was the highest among the biomarkers at 73.14%, indicating relatively better overall diagnostic accuracy compared to NLR, LMR, and PLR.

**Table 3:** Bivariate analysis for NLR, LMR, PLR, and CA-125 in distinguishing benign and malignant ovarian tumors.

	Cut-off	Sensitivity (%)	Specificity (%)	LR	AUC ROC (%)
NLR	≥ 2.72	68.29	67.47	2.099293	70.51
LMR	≥ 3.67	41.46	43.37	0.732121	33.86
PLR	≥ 174.1	65.04	65.06	1.838294	70.69
CA-125	≥ 78	67.48	68.67	2.153846	73.14

NLR: neutrophil-to-lymphocyte ratio; LMR: lymphocyte-to-monocyte-ratio; PLR: platelet-to-lymphocyte ratio.

### Bivariate analysis for The IOTA in distinguishing benign and malignant ovarian tumors

The performance of the IOTA in distinguishing between benign and malignant ovarian tumors was evaluated using bivariate analysis. The sensitivity of the IOTA in predicting malignancy was found to be 73.17%, indicating its ability to correctly identify a substantial proportion of malignant cases. However, the specificity was relatively lower at 38.55%, reflecting its limited capacity to correctly classify benign cases.

### Preliminary and final predictive model for malignancy in ovarian tumors using NLR, LMR, PLR, and CA125

The investigation into the predictive capacity of NLR, LMR, PLR, and CA-125 for ovarian tumor malignancy is detailed in Table 4. Multiple logistic regression was employed to analyze the P value of every respectable variable simultaneously. The study's results indicate that both PLR and CA-125 emerge as independent risk factors for malignancy in ovarian tumors, as evidenced by the statistically significant p-values (P < 0.05). Consequently, both PLR and CA-125 were incorporated into the final predictive model, as illustrated in Table 5. The final model reveals a positive correlation between elevated PLR and CA-125 levels and the presence of malignancy in a robust and simplicity model. Higher values of PLR and CA-125 correspond to an increased risk of malignancy in patients with ovarian tumors.

**Table 4:** Preliminary predictive model for malignancy in ovarian tumors using NLR, LMR, PLR, and CA-125.

	β	P (z)	β 95% CI		P (chi sq)	Pseudo R sq
NLR	0.11	0.19	− 0.05	0.27	0.00	0.17
LMR	− 0.15	0.21	− 0.38	0.08		
PLR	0.004	0.03	0.0004	0.01		
CA125	0.002	0.01	0.0004	0.003		
Constanta	− 0.66	0.35	− 0.205	0.73		

NLR: neutrophil-to-lymphocyte ratio; LMR: lymphocyte-to-monocyte-ratio; PLR: platelet-to-lymphocyte ratio.

**Table 5:** Final predictive model for malignancy in ovarian tumors using NLR, LMR, PLR, and CA-125.

Malignancy	β	P (z)	β 95% CI		P (chi sq)	Pseudo R sq
CA125	0.002	0.01	0.0004	0.003	0.00	0.15
PLR	0.006	0.00	0.003	0.009		
Constanta	− 1.36	0.00	− 2.08	− 0.65		

PLR: platelet-to-lymphocyte ratio.

### Nomogram model for predicting malignancy

**Figure 2:**
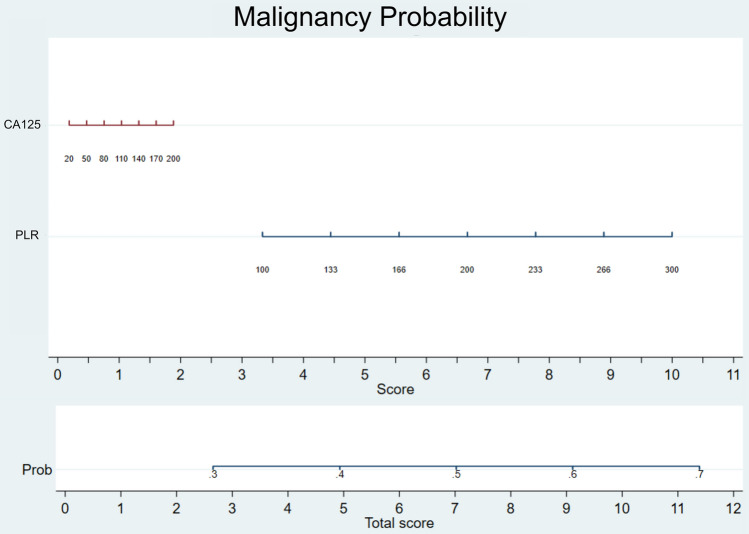
Nomogram for malignancy probability in ovarian tumor patients. PLR: platelet-to-lymphocyte ratio, CA125: cancer antigen 25, Prob: probability.

Utilizing the outcomes of multiple regression logistic analysis, a nomogram was meticulously constructed to predict malignancy in ovarian tumors, as shown in Fig. 2. The inclusion of PLR and CA-125 in the model reflects their identified roles as independent risk factors for malignancy, substantiated by the study's statistical findings. The value of significant variables is translated as the probability (risk) of malignancy. However, the probability is overestimated due to the coefficient produced by the logistic regression analysis. Therefore, use this nomogram for estimation of malignancy only. To determine the risk of malignancy, begin by obtaining the patient's values for CA-125 and PLR from laboratory tests. Locate these values on the nomogram scales provided for each biomarker. Draw vertical lines from each biomarker value to the points scale at the top of the nomogram, determining the points for each biomarker. Sum up the points obtained from all biomarkers to calculate a total score. Finally, refer to the risk scale at the bottom of the nomogram to find the corresponding probability of malignancy based on the total score.

## Discussions

Our findings highlight the potential of combining readily available hematological markers like NLR, LMR, and PLR with the established tumor marker CA-125 to improve the accuracy of ovarian cancer diagnosis. This study offers a novel contribution to the field of ovarian cancer diagnostics, particularly in the context of developing a predictive nomogram. Unlike the study by Guo et al., which developed a diagnostic nomogram incorporating serological and ultrasound findings for preoperative prediction of malignancy in patients with ovarian masses^9^, our approach focuses on serum biomarker CA-125 with inflammatory biomarkers such as NLR, PLR, and LMR. Additionally, our study builds on the findings of Huang et al., who demonstrated the combined utility of CA-125, NLR, and PLR in diagnosing borderline and malignant epithelial ovarian tumors^10^. Our research extends this combination by including the LMR, thereby offering a more comprehensive assessment of the inflammatory response associated with ovarian tumors. These parameters collectively form a predictive model aimed at enhancing the accuracy of distinguishing between benign and malignant ovarian tumors.

CA125 expressed on the surface of cells undergoing metaplastic differentiation into a Müllerian-type epithelium and released in soluble form into bodily fluids, serves as a valuable biomarker^5^. Our study affirms the continued relevance of CA125 in distinguishing between benign and malignant ovarian tumors, even considering its inherent limitations in sensitivity and specificity, particularly with a designated cut-off point of ≥ 78. In addition to its known limitations in sensitivity and specificity, the observed lower AUC values for CA-125 in our study underscore its challenges in effectively distinguishing between benign and malignant ovarian tumors. CA-125 elevation is not exclusive to ovarian cancer and can occur in other conditions such as endometriosis, smoking, and long-term oral contraceptive pill (OCP) use^11,12^. These factors can contribute to false positives and impact CA-125's diagnostic specificity. Additionally, the heterogeneity of ovarian cancer subtypes and stages. The effectiveness of using CA125 to differentiate between benign and malignant ovarian tumors also enhanced when combined with other biomarkers. A study has shown an increase in sensitivity and specificity when CA-125 is combined with glutaminase (GLS), compared to using CA-125 alone or GLS alone^6^.

Our study aimed to investigate the added value of hematological markers, such as NLR, LMR, and PLR, in diagnosing ovarian tumors, based on their roles in the immune response and inflammation. Leukocytes are critical components of both the innate and adaptive immune systems, comprising granulocytes, monocytes, macrophages, dendritic cells, and lymphocytes, which perform immune-stimulating or immunosuppressive functions^8^. In cancer patients, various pathways may be activated to suppress an effective adaptive immune response, enabling the tumor to evade immune cell destruction^13^. These leukocytes also release cytokines and growth factors that promote tumor growth, altering the blood leukocyte profile and serving as markers of the systemic inflammatory response^8^.

Imbalances in inflammatory markers have been linked to different cancers, highlighting their involvement in the pathophysiology of solid tumors^8^. Various measures of leukocyte quantities, such as WBC count, PLR, and NLR, have been associated with increased risk and progression of several cancers, including breast, colorectal, and endometrial cancers^8^. This indicates a broader applicability of these markers in cancer diagnosis and prognosis. However, their moderate AUC values reflect limited diagnostic accuracy as standalone tests, necessitating their combination with other markers like CA-125 to enhance diagnostic precision.

Our findings reveal a statistically higher NLR in malignant ovarian tumors compared to benign ones. This aligns with a study by Yilmaz, where they reported a significantly elevated NLR in ovarian cancer compared to benign cases. Furthermore, Yilmaz highlighted that pre-operative NLR in malignant patients was notably higher than post-operative NLR^14^. These observations suggest that NLR may serve as an indicator of malignancy and could potentially be associated with the dynamic changes occurring during the surgical and post-operative phases. Additionally, our findings resonate with another study's insights, indicating that a higher NLR is linked to incomplete tumor reduction, although not as an independent factor^15^.

Current study findings also underscore the role of LMR as a hematological marker in the discrimination of ovarian tumors. Specifically, our results indicate a notable association between lower LMR and malignant ovarian tumors. This aligns with a parallel study that reported a significant association between low levels of LMR and an increased risk of serous EOC compared with endometrioid EOC^16^. Moreover, our observations are reinforced by a meta-analysis, which demonstrated that a low LMR is associated with unfavorable survival outcomes in patients with ovarian cancer^17^.

Our study revealed that a cut-off point for PLR, specifically a value of ≥ 174.1, shown its potential utility in distinguishing between benign and malignant ovarian tumors. This aligns with findings from another study, where a slightly lower cut-off point of ≥ 150.2 was proposed for differentiating between benign/borderline and malignant ovarian tumors^18^.

However, the moderate AUC values NLR, LMR, and PLR reflect their limited ability to serve as standalone tests. The inflammation response and its role in carcinogenesis are complex, and these markers are influenced by a range of factors, including non-cancer-related inflammatory conditions such as infections, autoimmune diseases, chronic inflammatory diseases (e.g., rheumatoid arthritis, inflammatory bowel disease), and acute inflammatory responses (e.g., trauma, surgery)^19–21^. Additionally, cancer treatments such as chemotherapy or chemoradiation can also affect the levels of these markers^22^. To minimize these confounding effects, we collected blood samples from presurgical and before any treatment was administered. Furthermore, we excluded patients with infections, autoimmune diseases, and chronic inflammatory conditions to reduce the impact of these factors on the inflammatory and immune markers, ensuring a more accurate assessment of their diagnostic value.

The results from our study also highlight the diagnostic performance of several biomarkers and the IOTA in distinguishing between benign and malignant ovarian tumors. While the IOTA showed a sensitivity of 73.17%, its specificity was relatively low at 38.55%, which could lead to a significant number of false-positive results. This underscores the limitations of IOTA in accurately classifying benign tumors. In contrast, our study evaluated the diagnostic performance of individual inflammatory biomarkers, including NLR, LMR, PLR, and CA-125. Notably, CA-125 exhibited the highest AUC at 73.14%, suggesting better overall diagnostic accuracy compared to the other biomarkers. The combined use of PLR and CA-125 in our nomogram model demonstrated enhanced diagnostic performance. The PLR had a moderate discriminative capacity with an AUC of 70.69%, while CA-125 had a sensitivity of 66.67% and specificity of 68.67%.

Our study contributes to the field by constructing a nomogram model incorporating PLR and CA-125, recognizing both as independent risk factors for malignancy in ovarian tumors. The nomogram's development is informed by the non-normally distributed nature of CA-125 and PLR, prompting us to base the model on the distribution's first to third quartiles (or percentiles 25 to 75). This practical approach ensures a reliable and applicable nomogram despite the non-normative distributions of the data. Supporting our efforts, another study highlights the benefit of combining PLR with CA-125 to improve sensitivity in distinguishing between malignant and benign ovarian masses^23^. This evidence suggests that integrating PLR and CA125 in diagnostic assessments can enhance the accuracy of determining the malignancy status of ovarian tumors. In clinical practice, the integration of our nomogram with conventional diagnostic approaches can enhance the accuracy of malignancy risk assessment in patients with suspected ovarian tumors. This comprehensive approach ensures that patients identified as having a high probability of malignancy, as indicated by the nomogram, receive timely and appropriate management. Consistent with recommendations from authoritative bodies such as the National Comprehensive Cancer Network (NCCN) and the American College of Obstetricians and Gynecologists (ACOG), referral to a gynecologic oncologist for further evaluation and treatment is advised for all patients with suspected ovarian malignancies. This multidisciplinary approach has been associated with significantly improved survival outcomes due to the specialized expertise and tailored management strategies provided by gynecologic oncologists^24^.

Although our study results build on existing knowledge, they provide a meaningful contribution to the field of ovarian tumor diagnostics by highlighting the significance of combining CA-125 with NLR, LMR, and PLR. This approach enhances diagnostic accuracy and offers a practical and cost-effective method for differentiating between benign and malignant ovarian tumors. However, several limitations must be acknowledged: (1) Retrospective design, which have limitations regarding the quality and completeness of the dataset; (2) Non-normal distribution of CA-125 and PLR, it prompting us to base the nomogram model on quartiles. While this approach ensures practicality, it may introduce some level of approximation and may not fully capture the nuances of the biomarker distribution; (3) Our study is based on a specific cohort from our hospital, potentially limiting the generalizability of our findings to broader populations; (4) Small numbers of samples. The study population could also be larger, which may limit the generalizability of our findings. However, our study points towards future research opportunities. We suggest further studies to confirm and enhance the reliability of our nomogram model. Using larger groups of patients would help validate and enhance our predictive model.

In conclusion, our study underscores the significance of hematological biomarkers in diagnosing ovarian tumors. Specifically, higher levels of NLR, PLR, and CA-125 were consistently associated with malignant ovarian tumors, while lower LMR levels were indicative of malignancy. Furthermore, our findings highlight PLR and CA-125 as independent risk factors for distinguishing between benign and malignant ovarian tumors.

## Data Availability

The datasets used and analyzed during the current study are available in the corresponding author on reasonable request.
